# Enzyme-Assisted High Throughput Sequencing of an Expanded Genetic Alphabet at Single Base Resolution

**DOI:** 10.21203/rs.3.rs-3678081/v1

**Published:** 2023-12-21

**Authors:** Bang Wang, Kevin M. Bradley, Myong-Jung Kim, Roberto Laos, Cen Chen, Dietlind L. Gerloff, Luran Manfio, Zunyi Yang, Steven A. Benner

**Affiliations:** 1 Foundation for Applied Molecular Evolution, 13709 Progress Blvd, Alachua, FL, USA, 32615.; 2 Firebird Biomolecular Sciences, LLC, Alachua, FL, USA, 32615.; 3 Department of Chemistry, University of Florida, Gainesville, FL, USA, 32611.

**Keywords:** Sequencing, Expanded Genetic Alphabets

## Abstract

Many efforts have sought to apply laboratory *in vitro* evolution (LIVE) to natural nucleic acid (NA) scaffolds to directly evolve functional molecules. However, synthetic biology can move beyond natural NA scaffolds to create molecular systems whose libraries are far richer reservoirs of functionality than natural NAs. For example, “artificially expanded genetic information systems” (AEGIS) add up to eight nucleotides to the four found in standard NA. Even in its simplest 6-letter versions, AEGIS adds functional groups, information density, and folding motifs that natural NA libraries lack. To complete this vision, however, tools are needed to sequence molecules that are created by AEGIS LIVE. Previous sequencing approaches, including approaches from our laboratories, exhibited limited performance and lost many sequences in diverse library mixtures. Here, we present a new approach that enzymatically transforms the target AEGIS DNA. With higher transliteration efficiency and fidelity, this Enzyme-Assisted Sequencing of Expanded Genetic Alphabet (ESEGA) approach produces substantially better sequences of 6-letter (AGCT**ZP**) DNA than previous transliteration approaches. Therefore, ESEGA facilitates precise analysis of libraries, allowing ‘next-generation deep sequencing’ to accurately quantify the sequences of 6-letter DNA molecules at single base resolution. We then applied ESEGA to three tasks: (a) defining optimal conditions to perform 6-nucleotide PCR (b) evaluating the fidelity of 6-nucleotide PCR with various DNA polymerases, and (c) extending that evaluation to AEGIS components functionalized with alkynyl and aromatic groups. No other approach at present has this scope, allowing this work to be the next step towards exploiting the potential of expanded DNA alphabets in biotechnology.

## Graphical Abstract

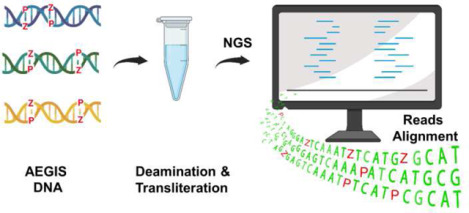

## Introduction:

A standard challenge in biotechnology arises from our inability to design molecules from first principles to meet the performance needed for biotechnological applications. Proteins, in principle, could deliver “performance on demand”; natural protein evolution does this for a spectacularly broad range of functions. However, computationally intensive protein design^1^ as well as protein-targeted laboratory evolution^2^ require enormous amounts of trial and error, as well as knowledge of thousands of pre-solved structures. Further, outside of privileged scaffolds (antibodies are exemplary), the enormous sequence space of proteins is dominated by molecules that do not fold or, worse, precipitate from water. Folding and dissolution in water are nearly universal requirements for biotechnological value.

Nucleic acids (DNA, RNA) have better-defined folding rules. Further, they remain soluble throughout their sequence spaces due to their repeating backbone charges^3^, and enjoy direct evolvability without the intermediacy of complex ribosome-based translation. RNA catalysts may have supported life during an episode of its early evolution, the “RNA World”^4^. Accordingly, pioneers like Larry Gold, Jack Szostak, Gerald Joyce, and others suggested that nucleic acids might be platforms for laboratory in vitro evolution (LIVE) to create functional biopolymers^5^.

Unfortunately, three decades of effort with LIVE on natural scaffolds have been often disappointing^6, 7.^ This disappointment has been attributed to the low information density of standard DNA/RNA (which hinders defined folding), their lack of functional groups needed for efficiently binding and catalysis, and the intrinsic difficulty of getting compact core folds from their polyanionic backbone.

**Fig. 1 |:**
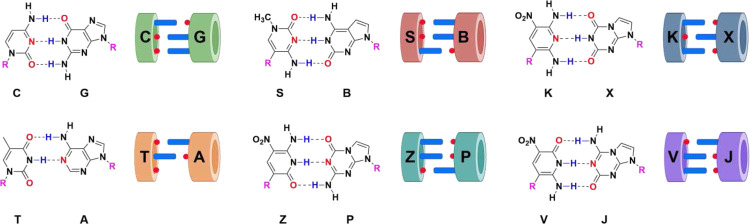
Chemical structures of standard and non-standard nucleobases. By rearranging hydrogen bonding donor and acceptor groups on base pairs in a Watson Crick geometry, the number of independently replicable informational units in DNA/RNA can be increased from 4 to 12, increase the information density, functionality, and density of binders and catalysts in libraries of oligonucleotides built from an artificially expanded genetic information system (AEGIS).

These limitations might be mitigated in DNA analogs that exploit alternative hydrogen bonding patterns to give “artificially expanded genetic information systems” (AEGIS, Fig. 1)^8^. For example, adding non-standard nucleobases adds alternative base-base interactions that dramatically expand the number of compact folds available to evolving AEGIS oligonucleotides. These include isoG pentaplexes^9^ (with the one letter code B), fat and skinny duplexes^10^, and the recently reported fZ-motif^11^. This last fold exploits the low pK_a_ of Z to give “skinny” deprotonated Z^−^: Z pairs in a novel parallel double helix.

Consistent with this, AEGIS-LIVE is proving to be a useful alternative to phage display and computationally intensive design for proteins, and as an alternative for systematic evolution of ligands by exponential enrichment (SELEX) for standard nucleic acids. Evolved AEGIS-bodies, antibody analogs, inactivate toxins^12^, bind cancer cell surface proteins^13, 14^, and deliver drugs selectively to targeted malignant cells^15^. AEGIS libraries from 6-nucleotide AEGIS DNA (G, A, C, T, **Z**, **P**, Fig. 1) are at least 100,000 times richer than standard GACT libraries as reservoirs for GACT**ZP** AEGISzyme ribonucleases, analogs of protein ribonucleases^16^. This is due to the ability of **Z** to act as a general acid-base catalyst. No comparable activity is seen with any standard nucleobase.

The challenge to support AEGIS-LIVE now is to develop methods that efficiently sequence 6-nucleotide (GACT**ZP**) AEGIS DNA. Since manufacturers of “next generation” sequencing instruments have not been persuaded to directly sequence non-standard AEGIS components of DNA, controlled transliteration of AEGIS DNA to standard DNA has been at the core of these methods.

Previously in these laboratories, a sequencing method was developed that integrates both an “easy” and a “difficult” transliteration^17^. The “easy transliteration” occurs when **Z:P** is converted to C:G through pairing between deprotonated Z and G. The **Z**^−^:G pair has a Watson-Crick geometry, allowing it to evade many proof-reading mechanisms. This makes it “easy”.

In contrast, a “difficult” transliteration requires T or C to pair opposite **P**. Neither is easy at standard PCR pHs, and therefore is not clean. This second transliteration means **Z:P** pairs are transliterated to a mixture of T:A and C:G pairs. The ratio in this mixture is very sensitive to condition, makes the bioinformatic analysis challenging, and preventing the analysis of very complex mixtures.

In collaboration with Andrew Laszlo teams, we have recently published preliminary data suggesting the possibility of using nanopores to sequence expanded genetic alphabets^18^. Similar approaches have been studied for hydrophobic unnatural nucleotides^19^. Nevertheless, these approaches remain in infancy.

Sequencing approaches of other unnatural nucleotide sets have suffered from similar challenges. For example, dye terminator Sanger sequencing^20^ with low throughput, the similar transliteration strategies^21–23^ were applied to hydrophobic pairs with NGS. Li and coworkers recently reported a clever transliteration strategy for Romesberg’s TPT3-NaM pair^24^. However, pairing between hydrophobic and hydrogen-bonding nucleobases, required for transliteration, need not always support quantitative sequencing results.

Thus, to fully realize the potential of AEGIS, we need reliable, efficient, quantitative, and user-friendly methods to sequence GACT**ZP** DNA. We report here such a method: Enzyme-Assisted Sequencing of Expanded Genetic Alphabet (ESEGA).

Here, rather than using transliteration during PCR, we enzymatically transform a starting mix to convert all cytidines to uridines using a member of the “Apolipoprotein B mRNA Editing Catalytic Polypeptide-like” (AID/ APOBEC^25^) deaminase family^26^. APOBeC converts standard cytidine (C) in an oligonucleotide to uridine (U), a deterministic transliteration that occurs in high yield.

Separately, we exploit the relatively low pK_a_ (≈ 7.8) of AEGIS **Z**, which in its deprotonated form mismatches with G. This allows clean transliteration of **Z:P** pairs to C:G pairs during PCR. Finally, since no standard nucleobase effectively mismatches with **P**, we developed a workflow that incorporates d**Z**TP into transliterative PCR to make the only necessary mismatch in the workflow to be between deprotonated **Z** and G.

Together, these allow us to exploit the power of next-generation sequencing (NGS)^27^ instruments. These deliver millions of reads from single samples for four-letter DNA. The final part of the workflow is bioinformatics. After deamination and 5-nucleotide PCR conversion, comparison of the results of deep sequencing of AEGIS PCR products, in parallel, with antisense and sense DNA, allows bioinformatics to infer the sequences of AEGIS-containing molecules in the starting mixture, even complex mixtures that arise from AEGIS-LIVE.

To demonstrate its utility, ESEGA was used to (1) Define optimal conditions to perform 6-triphosphate PCR conditions (such as buffer pH and d**P**TP concentrations). (2) Evaluate the 6-triphosphate PCR fidelity with various commercial and house-engineered DNA polymerases. (3) Extend that evaluation to functionalized AEGIS components, in particular, those with alkynyl and aromatic hydrophobic functional groups, which are sparsely introduced into AEGIS libraries because of the higher information density of a 6-letter GACT**ZP** DNA alphabet.

## Results:

**Fig. 2 |:**
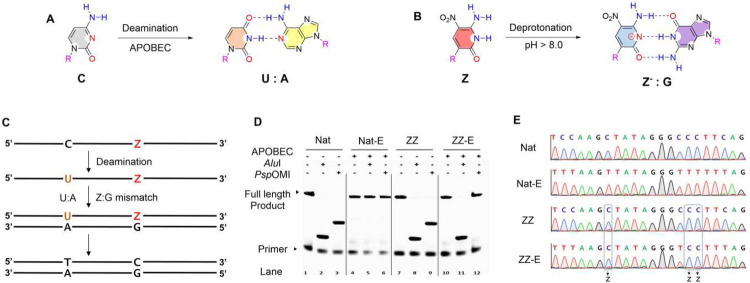
Sequencing 5-letter AEGIS (ATCGZ) DNA by deamination and transliteration. (A) Cytidine (C) is transliterated by cytidine deaminase to form uridine (U), which pairs to A in PCR. (B) AEGIS base **Z** becomes **Z**^−^ at pH (8.9) by deprotonation; **Z**^−^ pairs with G during PCR, inducing a Z to C transliteration. (C) Schematic workflow shows the C to T and **Z** to C conversions after deamination and PCR amplification. (D) Denaturing PAGE-urea analysis of restriction digestion of PCR products by TakaRa Taq DNA polymerase at pH8.9 from DNA templates (Nat and **ZZ**) without deamination or with deamination (Nat-E and **ZZ**-E). Forward primer was labeled by FAM at 5’. (E) Sanger sequencing demonstrates the precise transliteration of C to U (then T during the PCR) and Z to C.

To develop ESEGA sequencing, two single-stranded DNA sequences were synthesized to serve as test beds. These were accompanied by control sequences made from standard nucleotides (“Nat”), and a **Z**-modified sequence, where C was replaced by with **Z** in the natural sequence (Table 1). The “Nat” sequence contains two restriction sites that are recognized by two restriction endonucleases, Alul (AGCT) and PspOMI (GGGCCC). The **ZZ** trial sequence contains **Z**s placed strategically so that if they are transliterated to C, the Alul and PspOMI sites are re-generated (Supplementary Fig. 1). This transliteration can be detected by strategic restriction digestion (Fig. 2D).

To develop and metric ESEGA sequencing, samples of both Nat and ZZ sequences were treated with cytidine deaminase; controls were not treated. Then, treated and untreated sequences were PCR amplified (pH 8.9) in mixtures containing only four standard dNTPs (no d**Z**TP, no d**P**TP “4-triphosphate PCR”,). These conditions force template d**Z** to mis-direct incorporation of dGTP (Fig. 2B).

As the pH of PCR buffer may affect PCR efficiency, a series of pH values of PCR buffer (from 7.4–9.5, measured at room temperature) were evaluated by qPCR; C_q_ values were used as metrics. Both Nat and **ZZ** template were well amplified between pH 8.0 and 9.3 (Supplementary Fig. 4). As the preferred PCR conditions to facilitate **Z** → C transliteration, pH 8.9 was chosen. PCR products were then digested by Alul or PspOMI. The PCR amplicons from the natural template without deaminase treatment gave one well identified low length digestion band in denatured Urea-PAGE analysis in Lanes 2 and 3 (Fig. 2D), as expected from faithful amplification of the two sites in the synthetic standard DNA.

In contrast, PCR amplicons from the standard template that had been previously treated by deaminase (“Nat-E”) resisted restriction digestion (Fig. 2D, Lanes 5 and 6). This showed that the deaminase completely converted the Cs to Us in the restriction sites (Fig. 2A); these appear as T in the PCR amplicons (Fig. 2C).

When the ZZ template was amplified in PCR with just four standard triphosphates, amplicons were also well digested by endonucleases (Fig. 2D, Lanes 8 and 9). This showed that Z is converted to C during the PCR amplification. With ZZ template treated with cytidine deaminase (“ZZ-E”) and then amplified by PCR, amplicons were digested by AluI (Lane 11). This indicated that: (i) Z is not affected by cytidine deaminase; (ii) an isolated Z can be successfully transliterated to C.

However, the ZZ-E amplicons resisted the digestion by PspOMI (Fig. 2D, lane 12). This suggested that the PspOMI restriction site was changed from GGCZZ to GGTCC by deamination of the C to U and transliterating **ZZ** to CC. This also showed that C → U deamination by APOBEC was not affected by a neighboring **ZZ**.

To confirm this by Sanger sequencing, the length of the sequencing DNA was extended by tagged PCR (from 71 bp to 323 bp, Supplementary Fig. 2). The sequencing results (Fig. 2E) agree well with the restriction digestion. The sequence of the “Nat” amplicons matched with original design. The sequences of the Nat-E amplicons showed that all the Cs were deaminated. In the **ZZ** template, all of the **Z**s were transliterated to C by PCR (at pH 8.9). For the **ZZ**-E sample, the sequences showed that the original Cs were completely transliterated to Ts (Fig. 2E). However, they also show three Cs signals arising from the positions originally holding **Z**, either isolated **Z** or consecutive **ZZ**.

**Fig. 3 |:**
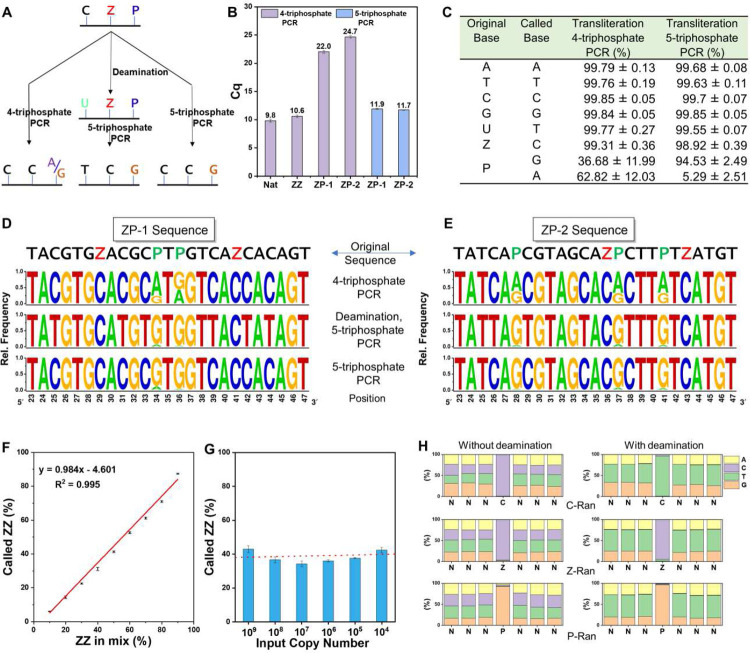
Enzyme-Assisted Sequencing of Expanded Genetic Alphabet (ESEGA) of single strand 6- letter DNA (ATCGZP). Complete NGS read summaries are in supplemental material. (A) Schematic diagram showing the transliteration of AEGIS (ATCG**ZP**) DNA using 4-triphosphate or 5-triphosphate PCR at pH8.9, without or with deamination. (B) Quantitative PCR (qPCR) to evaluate transliteration efficiency of various DNA templates in 4-triphosphate and 5-triphosphate PCR. Error bars represent the standard deviation of three independent experiments. Addition of d**Z**TP enhances the efficiency of PCR for 6-letter templates. (C) NGS data reveals the transliteration (%) of original bases [A, T, C, (U), G, Z, and **P**] in Nat, **ZZ**, **ZP**-1 and **ZP**-2 sequences under different PCR conditions (4-triphosphate or 5-triphosphate PCR at pH8.9, with or without deamination). Standard deviations represent the multiple bases in the three sequences. (D and E) Sequence logos of 6-letter DNA (**ZP**-1 and **ZP**-2) under different transliteration and PCR conditions. (F) Robustness and specificity of the ESEGA for **ZZ** and Nat sequences at different ratios (10%, 20%, 30%, 40%, 50%, 60%, 70%, 80%, and 90%). (G) Sensitivity of ESEGA for **ZZ** (45%) and Nat (55%) sequences in a series of 10-fold dilutions. The red dashed line represents the reference value obtained from Figure 3F. (H) Robustness of ESEGA with libraries NNNCNNN (C-Ran, top), NNN**Z**NNN (**Z**-Ran, middle) and NNNPNNN (**P**-Ran, bottom) sequences, without deamination (left panel) or with deamination (right panel), then, followed by 5-triphosphate PCR and NGS. These data show ESEGA has no strong context dependency.

We then investigated how DNA sequences built from six nucleotides “letters” (A, C, T, G, **Z**, **P**) were amplified under different PCR conditions. Two other test AEGIS DNA molecules were designed to contain both **Z** and **P** (**ZP**-1 and **ZP**-2, Table 1) and synthesized. For DNA sequences containing P to work when only standard (A, T, C, G) triphosphates are present, P is forced to mismatch with either C or T during the initial PCR cycles. However, this encounters problem with conversion of P, since all mismatches available in this amplification are incompatible with the Watson-Crick geometry (Fig. 3A, left arrow).

Isolated **Z**s and **P**s in **ZP**-1 were paired and read in primer extension experiments (Supplementary Fig. 3C). qPCR analysis showed that the C_q_ values of ZP-1 (C_q_ = 22.0) were higher than those of the Nat sequence (9.8) and **ZZ** sequences (C_q_ = 10.6), indicating the problematic nature of **P**:C and **P**:T mismatches (Fig. 3B). Further, when Z and P were adjacent (**ZP**-2), primer extension was completely inhibited (Supplementary Fig. 3D), and the C_q_ values of **ZP**-2 in the 4-triphosphate PCR were even higher (C_q_ = 24.7) (Fig. 3B). This suggest that P mismatching to T or C is more problematic when **Z** is adjacent.

This poor mismatching was mitigated by adding d**Z**TP to the four standard dNTPs (5-triphosphate PCR) (Fig. 3A right arrow). This allows **P** to match with **Z** in the first PCR round. The **Z** in its deprotonated form then directs the mismatched incorporation of G, leading to cleaner conversion. Thus, **ZP**-2 sequence work very well in 5-triphosphate primer extension (Supplementary Fig. 3E, F). Both **ZP**-1 and **ZP**-2 sequence show high efficiency in 5-triphosphate PCR, the Cq value (11.9 and 11.7) are close to Nat and **ZZ** template in 4-triphosphate PCR (Fig. 3B).

To obtain quantitative metrics for the fidelity of converting sequences built from 6-letter (A, C, T, G, **Z**, **P**) DNA to sequencable nucleotides under different PCR conditions, the performance of **ZP**-1 and **ZP**-2 conversion was compared with these pre-treatments:

(1) Direct amplification with 4-triphosphate PCR. This was expected to proceed with low efficiency with some ambiguous transliteration (Fig. 3A left arrow).

(2) Treatment with deaminase, followed by amplification with 5-triphosphate PCR. This was expected to deliver high efficiency PCR, with clean conversion of **Z:P** to C:G, with all of the C:G in the original templates replaced by U:A, and then T:A pairs (Fig. 3A central arrow)

(3) Direct amplification with 5-triphosphate PCR. This was expected to deliver high efficiency PCR, with clean transliteration of **Z:P** to C:G, and with all of the C:G in the original templates remaining as C:G pairs (Fig. 3A right arrow).

These amplicons were sent to Sanger sequencing (Supplementary Fig. 9 and 10) and NextGen sequencing. The analysis of NGS data revealed very faithful transliteration (>99%) of the original bases [ATC(U)G**Z**] in the Nat, **ZZ**, **ZP**-1, and **ZP**-2 sequences to their corresponding bases (A, T, C, and G) in 5-triphosphate or 4-triphosphate PCR. However, in the case of **P** transliteration, a mixture of A (~63%) and G (~37%) was observed in 4-triphosphate PCR, with relatively larger fluctuations. Fixing this problem, **P** was transliterated almost exclusively to G (~94.5%) in 5-triphosphate PCR (Fig. 3C). To visualize the transliteration of base at a given position, we converted the table information into a sequence logo (Fig. 3D and 3E), which illustrating the transliteration of **ZP**-1 and **ZP**-2 templates under for 4-triphosphate PCR (top), 5-triphosphate PCR following deamination (middle), and 5-triphosphate PCR without deamination (bottom).

As an innovative approach to test the robustness of ESEGA, **ZZ** and Nat templates were mixed with total concentrations of 100 nM, but with AEGIS-containing and standard oligonucleotides in various ratios (10%, 20%, 30%, 40%, 50%, 60%, 70%, 80%, and 90%). Subsequently, each sample underwent a single-stranded ESEGA with NGS.

The called populations of **ZZ** amplicons displayed a modest reduction of approximately 4.5% relative to the prepared **ZZ** percentage across all the templates (Fig. 3F).

This observation might be attributed to the slightly lower efficiency of **Z**^−^ mismatch with G in the first round of PCR compared with standard DNA base pair. This is supported by the higher C_q_ value recorded in qPCR for the **ZZ** template (10.6) in contrast to the Nat template (9.8) (Fig. 3B)

To evaluate the sensitivity of ESEGA, **ZZ** (45%) and Nat templates (55%) were blended and subsequently diluted serially to give six different concentrations and input to ranging from 10^9^ to 10^4^ copies in sequencing. Each sample was subjected to a single-stranded AEGIS-DNA sequencing. Sequencing was possible even at the highest dilution (Fig. 3G).

So far, the cytidine deamination and **Z/P** conversion were demonstrated with defined 6-letter DNA sequences. We were concerned that local sequence context might influence the outcome delivered by ESEGA. To determine whether neighboring nucleotides may affect C to U, **Z** to C, and **P** to G transliteration, three sequences were synthesized where a single C, **Z** or P was placed in the middle of six random nucleotides (C-Ran, **Z**-Ran and **P**-Ran, Table 1). The ESEGA workflow was applied. Low sequence bias was seen in the deamination results (Fig. 3H, top), consistent with the literature^28^. Further, no overall sequence context bias was observed in **Z** and **P** transliteration (Fig. 3H, middle and bottom).

**Fig. 4 |:**
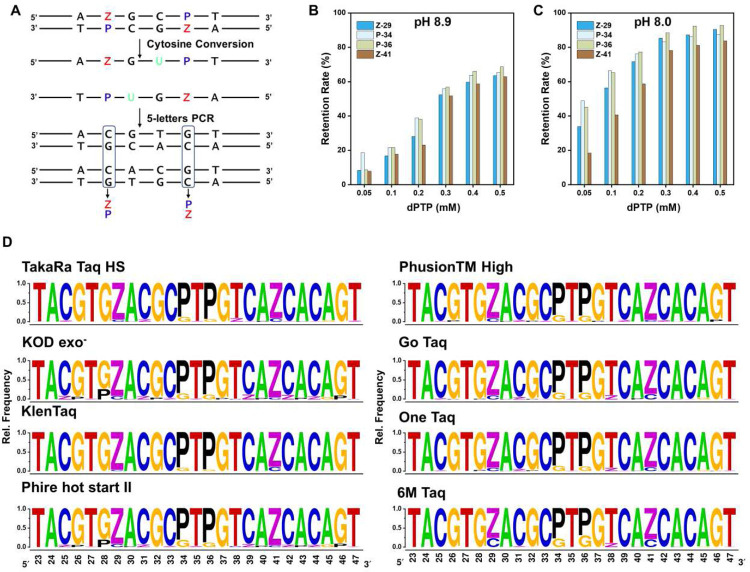
Sequencing double-stranded 6-letter DNA and assessing the fidelity of different DNA polymerases in 6-triphosphate PCR. (A) Schematic of ESEGA workflow for double-stranded 6-letter (ATCG**ZP**) DNA. (B) and (C) Retention rates of **Z/P** at each position in the **ZP**-1 template plotted after 6-triphosphate PCR with varying concentrations of d**P**TP (0.05–0.5 mM) at pH 8.9 or pH 8.0 conditions. (D) ESEGA evaluation of the fidelity of various polymerases in 6-triphosphate PCR. Complete NGS read summaries are in supplemental material.

Double-stranded DNA is a common outcome of AEGIS 6-nucleotide PCR. Thus, we developed a ESEGA workflow for double stranded DNA as well. First, the double-stranded DNA was denatured and the strands were separated. The two single-strands were separately treated with deaminase followed by 5-triphosphate PCR. The two amplicon pools were separately sequenced with barcode. Bioinformatics then matched the sequences to the strands that were originally paired. Then, the matches were analyzed to infer the original sequences of the paired strands (Fig. 4A). A:T and T:A pairs delivered A:T and T:A pairs in the duplexes matched by bioinformatics analysis, unchanged by the processes in the workflow. Thus, sites that hold A:T and T:A pairs in the surviving bioinformatics pairs were inferred to have been A:T and T:A pairs in the original duplex.

Likewise, **Z:P** and **P:Z** in the original duplex gave C:G and G:C in the bioinformatics-paired sites. In both cases, they arise by transliteration involving deprotonate **Z**:G mismatches. Thus, sites that hold G:C and C:G pairs in the surviving bioinformatics pairs were inferred to have been **Z:P** and **P:Z** pairs in the original duplex.

If the original duplexes have C:G or G:C pairs, then bioinformatics analysis gives a third outcome due to deamination. From amplicons arising from the strand that contained C, deamination gives amplicon duplexes with T:A pairs. From the complementary strand that contained G, the amplicon duplexes hold G:C pairs at the homologous site. Thus, bioinformatics assigns C:G in the original duplex when A:T appears in the amplicons derived from the “sense” DNA chain, if C:G appears also appears in amplicons derived from the anti-sense DNA chain.

### Applications of ESEGA

#### Determining 6-nucleotide PCR conditions that optimally retain Z:P pairs

To illustrate how ESEGA might be used in a practical setting, we first showed how ESEGA applied to double-stranded amplicons might be used to evaluate of various concentration of d**P**TP and various values of pH values the impact on the fidelity of GACT**ZP** PCR. Here, the metric for fidelity was the percent retention of **Z:P** pairs when the **ZP**-1 sequence was used as a template.

FAM-labeled forward and Cy5-labeled reverse primers were used to amplify **ZP**-1 in PCR with a complete set of six triphosphates, with a primer: template ratio of 50000:1 (~16 nominal doublings). TaKaRa Taq HS polymerase and 6-triphosphate dNTP (dATP (0.1 mM), dCTP (0.2 mM), dGTP (0.1 mM), dTTP (0.1 mM), d**Z**TP (0.1 mM), and d**P**TP) were used in the amplification. Six different dPTP concentrations (0.05 mM, 0.1 mM, 0.2 mM, 0.3 mM, 0.4 mM, and 0.5 mM) and two pH levels (8.0 and 8.9) were used, under the hypothesis that **Z:P** pairs would be better retained at higher concentrations of d**P**TP and better retained at lower pH (Supplementary Fig. 11).

Following separation of the PCR duplex amplicons by PAGE-urea, ESEGA was used to compare the data from the sense and anti-sense strands to quantitate the retention of the **Z** and **P** nucleotides after 25 rounds of PCR. Consistent with the hypothesis, **Z:P** pairs were better retained during PCR at pH 8.0 (Fig. 4C) than at pH 8.9 (Fig. 4B) at each concentration of dPTP. This was attributed to greater deprotonation of Z at the higher pH, leading to more deprotonated **Z**^**−**^**:G** mismatches. Greater retention of the **Z:P** pairs was also observed with increasing d**P**TP concentration. This was consistent with the hypothesis that d**P**TP competes with dGTP as a partner for template d**Z**.

Thus, ESEGA supported an application to screening PCR conditions to identify parameters that best retained **Z:P** pairs. Under these conditions, ~90% of the **Z:P** pairs were retained at pH 8.0 with 0.5 mM d**P**TP after 16 nominal doublings, with a nominal per cycle fidelity of 99.34%.

#### Identifying polymerases that optimally retain Z:P pairs

Using these optimal conditions (pH 8.0, 0.5 mM d**P**TP), we then amplify the **ZP**-1 template with a set of polymerases, including TaKaRa Taq HS, KOD exo^−^, KlenTaq, Phire hot start II, Phusion TM, Go Taq, One Taq, and an in-house-engineered 6M Taq variant. All of these gave amplification and interpretable sequencing data. The amount of each polymerase was adjusted to ensure similar amplification efficiency. Other polymerases examined (LongAmp Taq, Q5 High-Fidelity, Sulfolobus, Vent exo^−^, and HiFi KAPA) produced inconsistent results or no amplification at all.

ESEGA was used to analyze amplicons from 25 cycles of 6-triphosphate PCR using these eight polymerases and the **ZP**-1 template. The retention rates of **Z:P** pairs were visualized using a sequence logo (Fig. 4D).

Our findings revealed that **Z:P** pairs were retained best by the KlenTaq polymerase under these conditions, retaining 90–95% of the **Z:P** pairs after 16 nominal doublings; this approximates the uncertainty in the ESEGA analysis itself. However, the KlenTaq polymerase gave less efficient amplification. Thus, TaKaRa Taq HS was identified as a preferred enzyme under a metric that combined fidelity, efficiency, and robustness.

Additionally, we observed that KOD exo^−^ polymerase exhibited relatively good fidelity in the retention of **Z** and **P**, but added **Z** and **P** to the amplicons at positions that originated as C and G. The 6M Taq polymerase, which was developed in-house to encourage processivity, performed less well. A comprehensive description of the 6M Taq evolution process can be found in the Supplementary Materials. Overall, ESEGA provides a robust and reliable framework for the selection and development of high-fidelity polymerases in the context of 6-triphosphate PCR applications.

#### Assessing the fidelity of functionalized dPTP in 6-triphosphate PCR

As noted in the introduction, one of the advantages of AEGIS-LIVE over LIVE with standard nucleotides is the increased information density of expanded genetic alphabets, and the consequent ability to sparsely introduce functional groups in AEGIS-LIVE that standard DNA/RNA lacks. This allows AEGIS-LIVE to compete with protein evolution (e.g. phage display) and protein computational design (e.g. ROSETTA) by increasing the diversity of functional groups towards that of proteins, and increasing the number of compact folds, without the troublesome features of proteins, in particular, their propensity to precipitate.

**Fig. 5 |:**
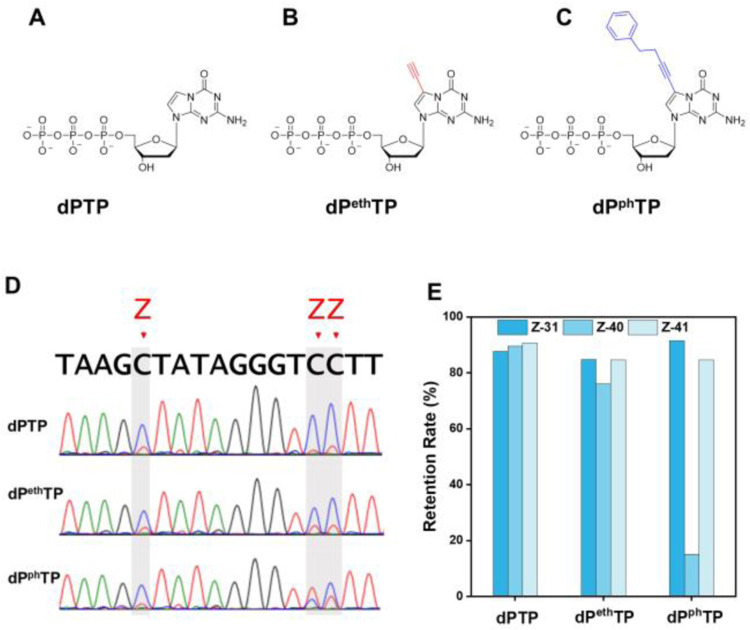
Chemical structures and retentions rate of functionalized-dPTP derivatives in 6-triphosphate PCR assessed by ESEGA. Chemical structures of (A) classical d**P**TP; (B) d**P**^eth^TP, 7-(4Ethynyl)-d**P** triphosphate; and (C) d**P**^ph^TP. 7-(4-Phenyl-1-butynyl)-d**P** triphosphate. (D) Sanger sequencing PCR amplicons from **ZZ** template with 6-triphosphate (dNTPs, dZTP, and dPTP, or dP^eth^TP, or d**P**^ph^TP). (E) ESEGA and NGS evaluate the retentions of d**P**, d**P**^eth^, and d**P**^ph^ in PCR amplicons after 6-triphosphate PCR by statistics of **Z** retentions.

Fig. 5B and Fig. 5C shows two variants of AEGIS P that carry functional groups, specifically, alkyl and phenylalkynyl groups. The first can support “click chemistry”; proteins have no analogous capability. The second is able to support hydrophobic interactions (compare with phenylalanine in proteins).

We used ESEGA to evaluate the performance of polymerases challenged to amplify AEGIS DNA containing these functionalized P variants. The **ZZ** sequence was chosen as template, with functionalized d**P**TP used in the triphosphate mix instead of normal d**P**TP. The amplification was done as before with 6-nucleotide PCR. The sense DNA chain was separated from resulting PCR products by PAGE-urea, and the sequences of the amplicons was evaluated by ESEGA, using both Sanger sequencing (Fig. 5D) and NGS (Fig. 5E). The 6-nucleotide PCR also monitored by qPCR (Eva green) when the fluor-labeled primers were replaced by unlabeled primers. (Supplementary Fig. 12B)

Here, the efficiency of amplification by TaKaRa of oligonucleotides containing alkynyl P was close to that of those with unfunctionalized **P**. Amplification efficiency was modestly lower with phenylalkynyl P (Supplementary Fig. 12B). The fidelity of replication was comparable with alkynyl **P** by ESEGA (Fig. 5E). However, substantial loss of phenylalkynyl P was observed by ESEGA, especially at position 40 of the template. Here, the two adjacent Zs drive the insertion of two tagged P’s. It is well known that unmodified polymerases such as TaKaRa do not easily synthesize DNA with two consecutive tagged nucleotides^29^. Thus, this result was not unexpected.

## Discussion

Nearly all life forms on Earth share the same informational biopolymers using A-T and C-G base pairs. Even the known exceptions, cyanoviruses that use diaminopurine instead of adenine as a partner for T, do not expand the number of independently replicable building blocks^30–32^.

However, standard nucleic acids lack the functional group diversity, the informational density, and the folding capability needed to give effective receptors, ligands, and catalysts. These factors account for the inability of laboratory *in vitro* evolution with standard DNA and RNA to compete effectively with antibody and laboratory protein evolution, even though proteins lacking a privileged scaffold are plagued by precipitation issues.

Artificially expanded genetic information systems (AEGIS, Fig. 1) are not designed for any specific purpose, but rather to be richer reservoirs of functionality in a directly evolvable system. AEGIS has more building blocks, a greater diversity of functional groups, higher information density, better control over folding, and the ability to form compact folds via base-base interactions. Even with the limited sequencing tools previously available, AEGIS-LIVE has evolved molecules that neutralize toxins, cleave specific RNA molecules, bind to specific cells, and deliver drugs to cancer tissues^33,34^. Alternative systems where pairing does not exploit inter-base hydrogen-bonding have been explored by Kool^35^, Hirao^22^, and Romesberg^36^. All have been shown to perform, at various levels of efficiency, in replication, transcription, translation and semi-synthetic organisms^8,37,38^. More exotically, AEGIS is helping us to seek alien life in the cosmos^39^, which may not have had the same pre-history as life on Earth, and thus may have different genetic biopolymers.

Therefore, AEGIS has the potential for broad biotechnological applications, should its evolution under selective pressures chosen by experimentalists become routine^40^. ESEGA offers a key element needed to make AEGIS-LIVE routine.

ESEGA represents a transformative use of the capabilities afforded by next generation sequencing, which has transformed the analysis of standard DNA sequences. By manipulating the pH level to alter the topological structure of nucleic bases, **Z** is deprotonated to form **Z**^−^, **Z**^−^ equates to C (Fig. 1). This allows **Z**^−^ to pair with G quite well, leading to high fidelity transliteration (99%). Additionally, ESEGA employs 5-triphosphate transliteration PCR to ensure high-fidelity transfer of P to G (~95%), This ensures a clean outcome.

Standard bioinformatics allow hundreds of thousands of reads from NGS to be analyzed in a library context. The cleanliness of this workflow supports high sequence diversity in those libraries, where each individual sequences present in the mix as only a few dozen exemplars.

In addition to showing this robust workflow, we show three applications where ESEGA supports the development of synthetic biology using expanded DNA alphabets. These are, of course, not the only three that can be conceived. Thus, ESEGA-like workflows hold the potential to develop other expanded genetic alphabets.

## Figures and Tables

**Table 1 |:** Standard and AEGIS DNA sequences used in this study.

Name	Sequence (5’−3’)
Nat	TAAGATGAGAGTTGAGGAGAGTTATCCAAGCTATAGGGCCCTTCAGTATAGTAGTGTAAGTAGATAGTGGA
ZZ	TAAGATGAGAGTTGAGGAGAGTTATCCAAGZTATAGGGCZZTTCAGTATAGTAGTGTAAGTAGATAGTGGA
ZP- 1	TAAGATGAGAGTTGAGGAGAGTTACGTGZACGCPTPGTCAZCACAGTATAGTAGTGTAAGTAGATAGTGGA
ZP- 2	TAAGATGAGAGTTGAGGAGAGTTATCAPCGTAGCAZPCTTPTZATGTATAGTAGTGTAAGTAGATAGTGGA
C-Ran	TAAGATGAGAGTTGAGGAGAGTTATNNNCNNNGTATAGTAGTGTAAGTAGATAGTGGA
Z-Ran	TAAGATGAGAGTTGAGGAGAGTTATNNNZNNNGTATAGTAGTGTAAGTAGATAGTGGA
P-Ran	TAAGATGAGAGTTGAGGAGAGTTATNNNPNNNGTATAGTAGTGTAAGTAGATAGTGGA

## ABBREVIATIONS

AEGIS: artificially expanded genetic information systems
LIVE: laboratory in vitro evolution
